# The Prevalence of Malaria among Children in Ethiopia: A Systematic Review and Meta-Analysis

**DOI:** 10.1155/2021/6697294

**Published:** 2021-04-13

**Authors:** Yalewayker Tegegne, Abebaw Worede, Adane Derso, Sintayehu Ambachew

**Affiliations:** ^1^Department of Medical Parasitology, School of Biomedical and Laboratory Sciences, College of Medicine and Health Sciences, University of Gondar, Gondar, Ethiopia; ^2^Department of Clinical Chemistry, School of Biomedical and Laboratory Sciences, College of Medicine and Health Sciences, University of Gondar, Gondar, Ethiopia

## Abstract

**Background:**

Malaria is one of the most public health important and life-threatening parasitic infections caused by the protozoan parasite. Since children are immunologically naive to the malaria parasite, they are the main vulnerable groups. During malaria infection, they might have a complication of anemia, cerebral malaria, coma, respiratory distress, and a decrease in cognitive and behavioral improvement. Therefore, this review was aimed at determining the pooled prevalence of malaria among children in Ethiopia.

**Methods:**

The current systematic review and meta-analysis were conducted based on the guideline of Preferred Reporting Items for Systematic Reviews and Meta-Analyses statement guideline. Electronic bibliographic databases such as Google Scholar, PubMed, and Science Direct were used for searching relevant literature. Besides, the Joanna Briggs Institute Meta-Analysis of Statistics Assessment and Review Instrument (JBI-MAStARI) was used for critical appraisal of studies. Using the STATA 14 software, the pooled Meta logistic regression was computed to present the pooled prevalence with a 95% confidence interval (CI).

**Result:**

The overall estimated pooled prevalence of malaria among children in Ethiopia was 9.07 (95% CI: 6.32, 11.82). Subgroup analysis based on malaria signs and symptoms showed that the pooled prevalence of malaria among asymptomatic and symptomatic children was 6.67% (95% CI: 0.36, 12.98) and 27.17% (95% CI: 18.59, 35.76), respectively.

**Conclusion:**

The findings revealed a high prevalence of malaria among children in Ethiopia. As a result, still there is a need of improving and rechecking the existing malaria prevention and control measures of the country.

## 1. Background

Malaria is one of the most important public health and life-threatening parasitic infections caused by the protozoan parasite. It is still a major concern in tropical and subtropical parts of the world. *Plasmodium falciparum*, *P. vivax*, *P. ovale*, *P. knowlesi*, and *P. malariae* are the five *Plasmodium* species that cause malaria in humans [1]. In 2018, the World Health Organization (WHO) report indicates that globally an estimated 228 million new cases of malaria were documented. The WHO African region accounted for 93% of all cases followed by the Southeast Asia region (3.4%) and Eastern Mediterranean region (2%). Likewise, there were an estimated 405000 deaths due to malaria in the globe. Children under the age of five-year were accounted for two-thirds (67%) of the world's malaria deaths in 2018 [2].

Around 75% of the landmass in Ethiopia is estimated to be malarious, and 68% of the total population in the area are at risk of malaria. According to the Ethiopian Federal Minister of Health (EFMH) report, malaria is on the list of the top ten leading causes of morbidity. *Plasmodium falciparum* accounts for 60%-70% of malaria cases, with the rest caused by *P. vivax*. The primary vector that plays an important role in malaria transmission is *Anopheles arabiensis*; moreover, *An. pharoensis*, *An. funestus*, and *An. nili* are less important vectors [3].

The EFMH indicates that around five up to six million malaria cases and tens of thousands of deaths of malaria were estimated each year. Moreover, in Ethiopia, malaria has a seasonal transmission and predominantly unstable, with frequent and often large-scale epidemics [4]. At this time, the diagnosis of malaria is by using microscopy or rapid diagnostic tests (RDTs) and treatment with artemisinin-based combination therapies (ACTs), promotion of long-lasting insecticidal nets (LLINs) ownership and use by the community, and application of indoor residual spraying (IRS) with insecticides were scaled up to improve access and equity to prevent and control malaria [5].

Since children are immunologically naïve to malaria parasites, they are the main vulnerable groups. They encounter severe clinical manifestation during *P*. *falciparum* infection. It may cause as many as 10% of all deaths in children [6]. Children during malaria infection might have a complication of anemia, cerebral malaria, coma, and respiratory distress and a decrease in cognitive and behavioral improvement [7]. Furthermore, despite the rapid administration of the best available antimalarial drugs, in sub-Saharan Africa, a minimum of one in ten children was admitted to hospitals as a result of severe and complicated malaria. Around 1200 children die from malaria in this region every day, accounting for the majority of the 631,000 deaths annually on the continent [8].

Different studies were conducted to assess the prevalence rate of malaria among children in Ethiopia having a great difference and varying findings. Besides, there is no previous systematic review and meta-analysis that assessed the prevalence of malaria among children in Ethiopia. Consequently, it was designed to assess the prevalence of malaria among children in Ethiopia. Its finding will support the execution of the most significant malaria prevention and control measure.

## 2. Methods

### 2.1. Searching Strategy

The current systematic review and meta-analysis were conducted by following the guideline of Preferred Reporting Items for Systematic Reviews and Meta-Analyses (PRISMA) statement [9]. The following electronic bibliographic databases were used for searching relevant literature: Google Scholar, PubMed, and Science Direct, to identify studies conducted on the prevalence of malaria among children. Only articles published in the English language up to August 2019 were retrieved. We used the search terms in correspondence with the Medical Subject Heading (MeSH) using the arrangement of keywords. In addition, the search terms were used separately and in combination using Boolean operators like “OR” or “AND”. The search strategy applied to retrieve important articles was as follows: (Prevalence) OR Prevalence [MeSH Terms]) AND Malaria) OR Malaria [MeSH Terms]) AND children) OR children [MeSH Terms]) AND Ethiopia) OR Ethiopia [MeSH Terms]. We had excluded duplicate data for the selection of the final studies included in this systematic review and meta-analysis. For managing references and remove repeated literature, the EndNote version X7 software was used.

### 2.2. Inclusion and Exclusion Criteria

#### 2.2.1. Inclusion Criteria

Primary research articles conducted to determine the prevalence of malaria among children in Ethiopia published in reputable peer review journal, studies used cross-sectional study design, and studies used malaria microscopy as a laboratory method were included. These studies could be conducted on symptomatic or asymptomatic children, studies either on health center and/or hospitals, or it could be studies on primary school or/and community-based studies.

#### 2.2.2. Exclusion Criteria

Small communication reports, review, poster, and studies reported on malaria prevalence by using only clinical signs and symptoms and studies used RDT as a laboratory method were excluded.

### 2.3. Search Methods and Quality Assessment

After we had searched the published research papers, it was imported into the EndNote X7 version, and then, duplicates were removed. All the authors (YT, SA, AD, and AW) had separately screened literature by their title, abstract, and full text to identify potentially appropriate articles according to the preset inclusion criteria. Then, the quality of all the incorporated studies was appraised by using the Joanna Briggs Institute (JBI) quality assessment checklist tool [10]. By looking at abstracts and titles, five hundred sixty-seven research articles were checked for eligibility criteria. Ninety-seven of them were selected for full-text evaluation by all of the authors. The discrepancy in choosing research papers to be included in this systematic review and meta-analysis were resolved by decision; in fact, we did have a very limited discrepancy between the authors in the selection of literature.

### 2.4. Data Extraction

All the authors of this paper came together and established the data extraction form in Microsoft Excel Spreadsheet. This data extraction sheet included the name of the first author, year of publication, study population, study design, study period, sample size, sampling technique, diagnostic method, the overall prevalence of malaria, the prevalence of *P. vivax*, the prevalence of *P. falciparum*, and the prevalence of mixed infection. Finally, the extracted data files were scientifically checked for reliability, and the existence of any discrepancies between the extracted data was resolved by detailed discussion among authors.

### 2.5. Data Analysis

Once we had extracted the data by using Microsoft Excel, the Stata version 14 software was used for statistical analysis. We use random-effects models as there is high heterogeneity across the study. Otherwise, it produces study encumbrances that principally indicates between study variation [11]. Basically, the *I*^2^ statistics predicates the existence of the main difference between studies included in a systematic review and meta-analysis due to heterogeneity rather than by chance, and its value range from 0 to 100%. *I*^2^ values of 25%, 50%, and 75% denote low, medium, and high heterogeneity between studies, respectively [12]. The existence of heterogeneity was determined when a *p* value was less than 0.05. Furthermore, we had checked the presence of publication bias, conducted subgroup analysis and sensitivity analysis.

## 3. Result

### 3.1. Characteristics of the Included Studies

**Figure 1:**
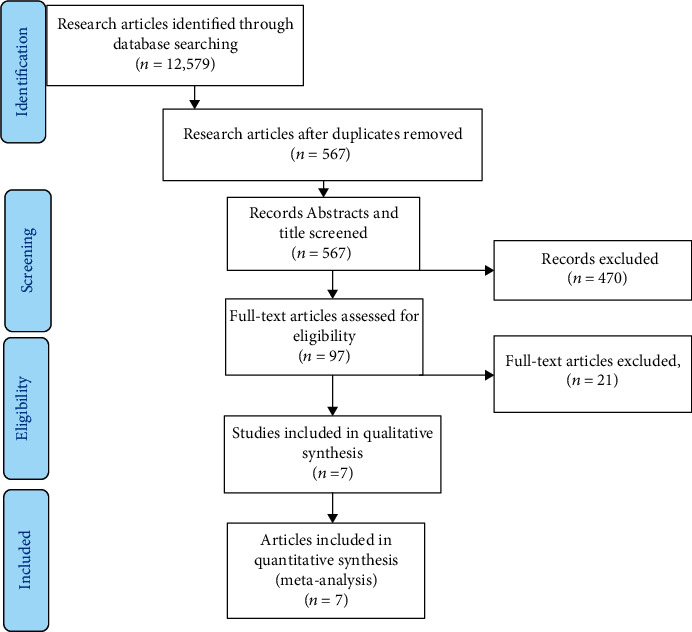
The flow diagram refers to the selection of studies included for a systematic review and meta-analysis of the prevalence of malaria among children.

By considering all the inclusion and exclusion criteria, a total of seven published studies on the prevalence of malaria among children were included. Initially, 12,579 published articles were identified via a database search engine. After that, 12,012 duplicates were removed from the total 12,579 searched research papers. Then, five hundred sixty-seven studies were screened by their title and abstract. Finally, after we had evaluated the full text of ninety-seven studies, seven of them were found to be eligible for this systematic review and meta-analysis. Full steps of screening and eligibility and the number of research articles were selected at each step as described in the diagram below (Figure 1). With regard to the type of study design used in the included studies, all of them utilized cross-sectional study design. On the other hand, from all the included studies, a study conducted in the Benishangul Gumuz region, Ethiopia, had the least sample size with 263 study participants [13]. However, another study conducted in the Oromia region, Ethiopia, had the highest sample size of 20,899 [14]. Microscopic diagnosis of malaria was the type of laboratory method used by all of the included studies. Also, most of the studies were from the Oromia region [14, 15], followed by the South Nation Nationality and People of Ethiopia [16, 17], and a single study was obtained from each region of Amhara and Benishangul Gumuz [13, 18]. But no studies were reported from the rest regions of Ethiopia.

In the current systematic review and meta-analysis, the total number of children included were 26,148. We had classified the included studies based on the type of cases so that most of the articles were conducted among children with asymptomatic malaria 4 (57%). Whereas 3 (43%) of the included studies were conducted among children with symptomatic malaria. In this systematic review and meta-analysis, most of the regions were included. The JBI quality assessment tool was used for assessing the quality of all the studies included in this systematic review; as a result, all of them have a good quality (Table 1).

### 3.2. The Prevalence of Malaria among Children

**Figure 2:**
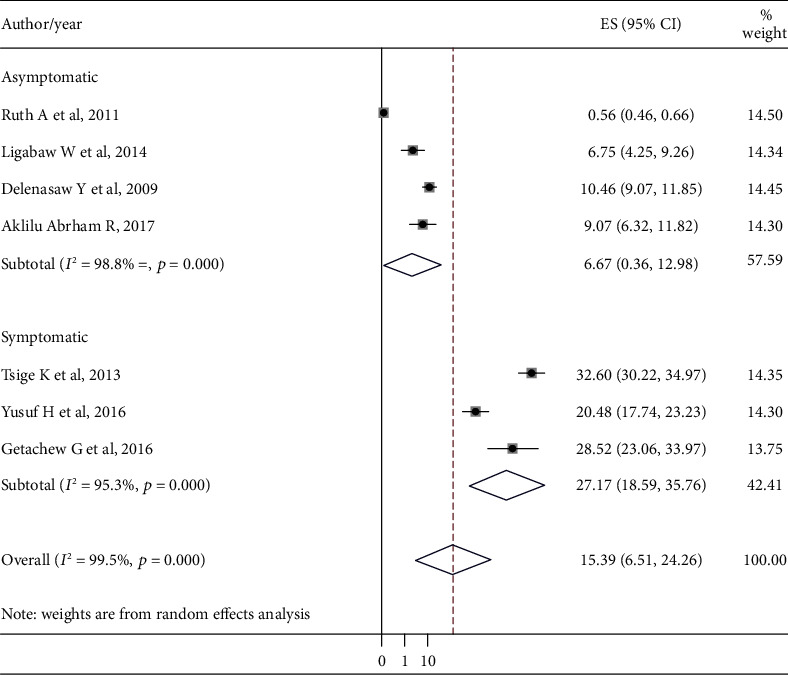
The pooled prevalence of malaria among children from random-effects model.

By including the seven published research articles, we had estimated the prevalence of malaria among children in Ethiopia. Accordingly, the overall estimated pooled prevalence of malaria among children was 9.07 (95% CI (6.32, 11.82)). The prevalence of malaria in each study as well as the pooled estimate was indicated by forest plot. According to the subgroup analysis, based on the existence of malaria symptoms, the prevalence of malaria among asymptomatic and symptomatic children was 6.67% (95% CI: 0.36, 12.98) and 27.17% (95% CI: 18.59, 35.76), respectively (Figure 2).

## 4. Discussion

Basic information is generated in this systematic review and meta-analysis about the estimated pooled prevalence of malaria among children in Ethiopia. In endemic areas, the risk of severe malaria is high among children. Despite symptoms of severe malaria such as severe anemia and respiratory distress occur at all ages, anemia and hypoglycemia are particularly common in young children [19]. Besides, in Africa, approximately 20 percent of all child deaths are caused by malaria. Certain children may have an acute attack of cerebral malaria that rapidly proceeds to coma and death [20].

The estimated combined pooled prevalence of malaria among children in Ethiopia in this systematic review and meta-analysis was 15.39 (95% CI: 6.51, 24.26). The result of this systematic review and meta-analysis was much higher than the recent national prevalence of malaria among the general population in Ethiopia. Parasite prevalence in Ethiopia was 0.5% by microscopy and 1.2% by RDTs for areas below 2,000 meters and less than 0.1% prevalence above 2,000 meters [21]. This might be due to the fact that children are a group of people with low immunity and immunity for them developed over years of exposure so that most malaria cases and deaths occurred among children [22].

Moreover, the result of this systematic review and meta-analysis was also higher when compared with meta-analysis done in sub-Saharan African countries 1.44% [23]. The high prevalence of malaria in Ethiopia as compared with countries in sub-Saharan Africa might be due to differences in the insecticide-treated bed nets, the type of test method used, the difference in the climatic conditions, and inadequate treatment of children with malaria cases. So that this systematic review and meta-analysis indicate for better decrement of malaria burden among children the world health organization malaria prevention and control strategies such as applying enhanced case management, and wide use of long-lasting insecticidal nets (LLINs) and indoor residual spraying (IRS) and early diagnosis and treatment and environmental managements should always be practiced in Ethiopia.

Surprisingly, a wide variation was observed in the prevalence of malaria among children in the current systematic review and meta-analysis compared with different national studies conducted in East Africa such as Uganda 19.7% [24], Rwanda 5.5% [25], and Kenya 16.27% [26]. The variation in the prevalence of malaria might be related to the difference in the type of laboratory method used, time of the study, and the difficulty of implementing the existing malaria prevention and control measures practice.

According to the subgroup analysis carried out in this systematic review and meta-analysis, the prevalence of malaria was found to be higher among children with malaria signs and symptoms 27.7% compared with asymptomatic children 6.67%. The high prevalence of malaria among symptomatic children might be related to the level of malaria parasitemia, and the chance of detecting malaria parasites is mostly higher whenever patients had signs and symptoms like fever.

The studies incorporated in this systematic review and meta-analysis showed a high heterogeneity. Despite all the authors involved in this review have tried to assess the possible sources of heterogeneity via subgroup analysis, the sources of inconsistency were not recognized. The possible source of heterogeneity might be related to the seasons in which each of the included studies was conducted because some of the studies were conducted during the high malaria transmission seasons, whereas the rest of included studies were done during the low malaria transmission seasons.

## 5. Conclusion

In conclusion, compared with the national prevalence of malaria among all the age groups, the estimated pooled prevalence of malaria among children was found to be higher. As a result, still, there is a need of improving and rechecking the existing malaria prevention and control measures of the country.

## Figures and Tables

**Table 1:** The included studies general characteristics in the systematic review and meta-analysis of the prevalence of malaria among children in Ethiopia, 2020.

Author/year	Region	Type of study design used	Type of cases	Type of laboratory method	Sample size	Prevalence of malaria (%)	Study quality
Ruth A et al., 2011	Oromia	Cross-sectional	Asymptomatic	Microscopic	20899	0.56	Good
Ligabaw W et al., 2014	Amhara	Cross-sectional	Asymptomatic	Microscopic	385	6.75	Good
Tsige K et al., 2013	SNNP	Cross-sectional	Symptomatic	Microscopic	1497	32.59	Good
Yusuf H et al., 2012	Oromia	Cross-sectional	Symptomatic	Microscopic	830	20.48	Good
Delenasaw Y et al., 2009	Oromia	Cross-sectional	Asymptomatic	Microscopic	1855	10.46	Good
Getachew G et al., 2016	Benishangul Gumuz	Cross-sectional	Symptomatic	Microscopic	263	28.52	Good
Aklilu R, 2017	SNNP	Cross-sectional	Asymptomatic	Microscopic	419	9.07	Good

## Data Availability

In this systematic review and meta-analysis, the main part of the data generated or analyzed during this study is included. Additional relevant data will be available from the corresponding author upon the need.

## Abbreviations

EFMH: Ethiopian Federal Minister of Health
IRS: Indoor residual spraying
JBI: Joanna Briggs Institute
LLINs: Long-lasting insecticidal nets
PRISMA: Preferred Reporting Items for Systematic Reviews and Meta-Analyses
RDTs: Rapid diagnostic tests
WHO: World Health Organization.
